# Tualang Honey: A Decade of Neurological Research

**DOI:** 10.3390/molecules26175424

**Published:** 2021-09-06

**Authors:** Khairunnuur Fairuz Azman, Che Badariah Abd Aziz, Rahimah Zakaria, Asma Hayati Ahmad, Nazlahshaniza Shafin, Che Aishah Nazariah Ismail

**Affiliations:** 1 Department of Physiology, School of Medical Sciences, Universiti Sains Malaysia Health Campus, Kubang Kerian 16150, Malaysia; khairunnuur@usm.my (K.F.A.); badariah@usm.my (C.B.A.A.); rahimah@usm.my (R.Z.); asmakck@usm.my (A.H.A.); 2 Brain and Behaviour Cluster (BBC) School of Medical Sciences, Universiti Sains Malaysia Health Campus, Kubang Kerian 16150, Malaysia

**Keywords:** Tualang honey, antioxidants, nootropics, antinociceptive, antidepression, anxiolytics

## Abstract

Tualang honey has been shown to protect against neurodegeneration, leading to improved memory/learning as well as mood. In addition, studies have also demonstrated its anti-inflammatory and antioxidant properties. However, a substantial part of this research lacks systematization, and there seems to be a tendency to start anew with every study. This review presents a decade of research on Tualang honey with a particular interest in the underlying mechanisms related to its effects on the central nervous system. A total of 28 original articles published between 2011 and 2020 addressing the central nervous system (CNS) effects of Tualang honey were analysed. We identified five main categories, namely nootropic, antinociceptive, stress-relieving, antidepressant, and anxiolytic effects of Tualang honey, and proposed the underlying mechanisms. The findings from this review may potentially be beneficial towards developing new therapeutic roles for Tualang honey and help in determining how best to benefit from this brain supplement.

## 1. Introduction

Malaysian honey is classified according to the bee species, or the floral sources of the honey [1]. There are two main types of bee species, namely Apis (*A. dorsata*, *A. mellifera* and *A. cerana*) (stinging bee) or Meliponine (stingless bee; locally known as Kelulut) [2]. According to floral sources, honey is further classified into monofloral (Acacia honey, Gelam honey, Pineapple honey, Leaf honey, Durian honey, Melaleuca honey, Coconut honey, Starfruit honey and Wax apple honey) or polyfloral honey (Tualang honey, Kelulut honey). An example of extra-floral honey is Rubber honey [3]. 

**Figure 1:**
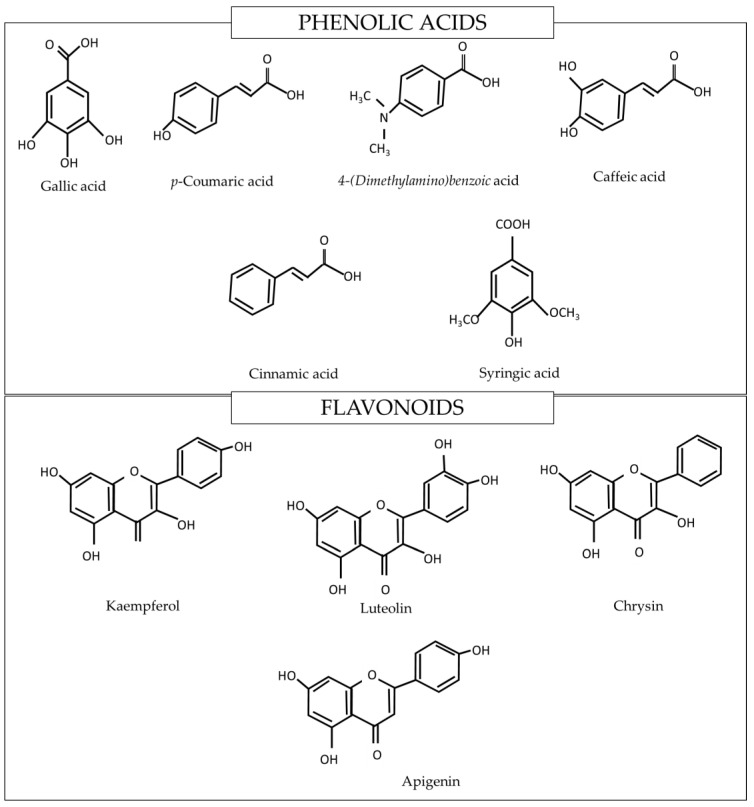
Some of the phenolic compounds found in Tualang honey [11].

Tualang honey is a wild polyfloral honey produced by *Apis dorsata*. Tualang honey has a dark brown appearance, a pH of 3.6–4.0 with a specific gravity of 1.34 [4]. It is slightly more acidic than other local Malaysian honey, such as Kelulut and Gelam [5], but its low pH is similar to Manuka honey [6]. The sugar composition of Tualang honey is mainly composed of monosaccharides, such as fructose (41.73%) and glucose (47.13%), and disaccharides, such as sucrose (1.02%) and maltose (4.49%) [7]. Several types of phenolic acids (gallic, coumaric, syringic, caffeic, cinnamic, benzoic, chlorogenic, salicylic and ferulic acid) and flavonoids (catechin, quercetin, kaempferol, luteolin, hesperetin, apigenin, 3,7,4′-trihydroxyflavone, naringenin, chrysin, fisetin, vitexin, isoorientin, xanthohumol pinobanksin-3-o-propionate and pinobanksin-3-o-butyratengenin) have been identified in Tualang honey [8,9,10]. Tualang honey contains some common phenolic compounds as found in other honey (Figure 1) [11]. 

Tualang honey’s properties are comparable to other types of honey (Table 1). Interestingly, Tualang honey contains more phenolic acids and flavonoids compared to Manuka and other local Malaysian honey [12] and is also more effective against some of gram-negative bacteria [13].

Honey has been used in traditional medicine since 2100 BC [14]. The Mayans, Babylonians, Romans, Egyptians, Chinese, and Greeks all consumed honey for its nutritional and therapeutic characteristics [15]. Most health advantages ascribed to honey have been anecdotal, based on observations and generalisations with little scientific backing. However, in the last decade, there has been a revived interest in researching honey’s possible health advantages. Moreover, honey has antioxidant, antibacterial, anti-cancer, anti-inflammatory, antidepressant, anxiolytic, and anti-stress properties [16]. Previous reviews on Tualang honey showed comparative differences in medicinal properties [13], potential anti-cancer properties [17], and physicochemical properties [18]. Although the potential roles of honey and honeybee products in neurological actions [19] as well as in learning and memory have been reviewed [20], other potential neurological effects, particularly of Tualang honey, remain to be comprehensively reviewed. This article highlights the current literature on the medicinal effects of Tualang honey with a special focus on its neurological effects based on the mechanisms identified. The possible underlying mechanisms of its effects and its future applications are also discussed.

## 2. Results and Discussion

A total of 28 papers published between 2011 to 2020 were analysed. We have identified five main categories, namely nootropic, antinociceptive, stress-relieving, antidepressant, and anxiolytic effects of Tualang honey, to discuss and propose the possible underlying mechanism.

### 2.1. Nootropic Effects of Tualang Honey

The nootropic effects of Tualang honey have been investigated in greater detail in animals than in human. Tualang honey was originally found to exhibit nootropic qualities in humans in 2011, and in experimental animals in 2014. In postmenopausal women, Tualang honey supplementation for 16 weeks was able to improve immediate memory and reduce oxidative stress levels comparable with the improvement seen in women receiving oestrogen plus progestin therapy [21,22,23]. Another clinical study on Tualang honey was recently conducted in schizophrenia patients whereby eight weeks of Tualang honey supplementation improved total learning performance across domains in the immediate memory, but not in long-term memory [24]. This recent study proved a promising role of Tualang honey as a cognitive enhancer in humans.

Tualang honey reduced uterine and vaginal atrophy in ovariectomised rats [25], qualifying it to be considered a phytoestrogen. Phytoestrogens are phytochemicals that are structurally similar to mammalian oestrogens and can therefore bind to oestrogen receptors, eliciting either oestrogenic or anti-oestrogenic effects depending on certain factors such as their concentration [26]. Some flavonoids such as chrysin, genistein, naringenin and luteolin exhibit oestrogenic activity and are also often referred to as phytoestrogens [27]. Phytoestrogens possess a neuroprotective effect [28] and have been shown to improve the memory domains of cognition as well as executive function [29,30]. Phytoestrogens are also able to substitute for oestrogen in elderly individuals with Alzheimer’s disease as well as depleted oestrogen levels [31]. Besides that, phytoestrogens are excellent antioxidants [32]. Thus, the nootropic effects of Tualang honey as evidenced in postmenopausal women [21,22,23] and schizophrenic patients [24] are most likely due to their high contents of phenolic compounds, such as chrysin, naringenin, and luteolin, that exhibit antioxidant as well as oestrogenic activities [33]. 

The nootropic effects of Tualang honey, using a similar dose of 200 mg/kg body weight of Tualang honey supplementation for 18 days, in stressed ovariectomized rats was able to enhance their short- and long-term recognition memory comparable to those receiving 17β-oestradiol treatment [34]. Ovariectomy is an animal menopausal model, associated with depleted oestrogen levels and cognitive ageing [35]. Stress and stress hormones such as corticosterone may impair long-term potentiation (LTP) and neurogenesis while enhancing long-term depression (LTD) in the hippocampus, consequently resulting in stress-induced memory deficits [36]. The combination of stress and ovariectomy significantly result in higher corticosterone levels (6000 pg/L) with short- and long-term memory deficits that are significantly correlated with a reduction in hippocampal neuronal number [34]. Following treatment with Tualang honey, the corticosterone levels were found to be reduced [37], while neuronal proliferation in the medial prefrontal cortex [38] and hippocampal CA2, CA3, and DG regions was enhanced [34]. These mechanisms may underlie the memory improvement observed in the Tualang honey treated rats [14]. As a phytoestrogen, Tualang honey exhibits its oestrogenic activity possibly by activating oestrogen receptor-mediated cell survival signalling pathways, thus promoting neuronal survival in the hippocampus and preserving memory function.

Similarly, in adult male rats exposed to noise stress, 35 days of Tualang honey supplementation at 200 mg/kg body weight significantly improved their short- and long-term recognition memory [39]. Stress exposure significantly induced memory impairment associated with oxidative stress in the brain and decreased neuronal density in the medial prefrontal cortex and hippocampal CA2 and CA3 regions [40,41]. Tualang honey supplementation significantly decreased levels of corticosterone and ameliorated the brain antioxidant status as well as the medial prefrontal cortex and hippocampal morphology [40,41]. 

Furthermore, Tualang honey possesses protective effects against memory deterioration due to ageing [40]. Ageing leads to a progressive loss of cognitive function associated with decreased function of cholinergic neurons in the hippocampus and cortex, whereby the survival and functional maintenance of the cholinergic neurons are dependent upon nerve growth factor (NGF) and brain-derived neurotrophic factor (BDNF) [42]. Supplementation with Tualang honey to naturally aged male rats significantly improved memory function as well as the morphology of the medial prefrontal cortex and hippocampus as manifested by the increased number of neuronal cells [40]. Moreover, Tualang honey treated rats exhibit significantly higher BDNF concentration and lower brain acetylcholinesterase levels [42]. Flavonoids have been demonstrated to enhance memory function via activating the extracellular signal-regulated kinase (ERK1/2) and protein kinase B (PKB/Akt) signalling pathways, leading to the activation of cAMP response element-binding protein (CREB), a transcription factor responsible for increasing BDNF expression [43]. Hence, we hypothesise that the memory preserving and neuroprotective effects of Tualang honey are due to its flavonoid contents acting on the ERK-CREB-BDNF pathway.

Aside from ageing, Tualang honey exerts its memory-enhancing effects in young and adult animals as well [44]. Supplementation of Tualang honey at a dose of 70% for 12 weeks to 7–8 weeks old male rats significantly enhanced learning and memory compared to control rats receiving 0.9% saline [44]. The Tualang honey treated rats exhibited a significantly higher number of pyramidal neurons in the CA1 and CA3 hippocampal regions [44]. A subsequent study revealed that Tualang honey exerted morphometric effects on the hippocampal CA1 pyramidal neurons whereby the somatic size of the CA1 neurons was significantly bigger and had less roundness than controls receiving saline [45]. The larger soma may indicate larger cellular and metabolic systems of the neuronal dendritic tree, more synaptic connections, and higher neuronal activities [46], possibly leading to enhanced memory ability. The lesser roundness of the soma reflects better retention of the pyramidal shape of the hippocampal neurons [45]. Therefore, these morphological findings suggest the neuroprotective effect of Tualang honey on the hippocampal neuronal soma indicative of enhanced memory and learning ability.

Tualang honey has also been reported to mitigate memory impairment induced by hypoxia [47] and lipopolysaccharide [48]. Hypoxia exposure may cause oxidative stress, alteration in cholinergic and glutamate neurotransmissions, as well as neuronal apoptosis in the cortex, striatum, and hippocampal cells, ultimately causing memory impairment [49,50]. Tualang honey administration at 200 mg/kg body weight for 14 days to hypoxia-induced rats resulted in significant improvement in spatial and recognition memory as well as reduced neuronal damage in the hippocampus compared to hypoxia-induced rats treated with sucrose [47]. In lipopolysaccharide-induced rats, a rat model of Alzheimer’s disease, a similar dose and duration of Tualang honey treatment significantly improved spatial and recognition memory comparable to the group treated with memantine, a medication used to treat Alzheimer’s disease [48]. It was also discovered that 150 mg/kg of methanolic fraction of Tualang honey produced similar effects [48]. Further investigation revealed that Tualang honey and its methanolic fraction exhibited significant neuroprotective effects against oxidative stress, hippocampal neurodegeneration, and amyloid deposition in the lipopolysaccharide-induced rats, although pure Tualang honey exhibited slightly higher protective activity than its methanolic fraction [51]. It was suggested that the pure honey composition together produced synergistic effects exhibited as higher neuroprotective potential compared to the methanolic fraction which contains only the non-polar compounds such as flavonoids and phenolic acids. Nevertheless, the polyphenolic compounds either in pure Tualang honey or its methanolic fraction are most possibly the ones responsible for the protective effect against lipopolysaccharide-induced amyloid deposition. Lipopolysaccharide induces chronic inflammation and oxidative damage in the brain resulting in the production and aggregation of beta-amyloid (Aβ) in both the hippocampus and cerebral cortex [51,52,53]. Polyphenolic compounds found in honey such as gallic acid have been shown to reduce Aβ1−42 aggregation and prevent the formation of toxic oligomers and fibrils in cultured primary hippocampal neurons by improving intracellular calcium influx [54]. Apigenin has been shown to affect amyloid precursor protein (APP) by processing and preventing Aβ deposition due to the downregulation of the β-site APP cleaving enzyme 1 (BACE1) in transgenic mice [55]. Thus, it is speculated that these polyphenolic compounds in Tualang honey also express anti-amyloidogenic properties.

In a rat model of chronic cerebral hypoperfusion-related neurodegenerative diseases, permanent bilateral occlusion of the common carotid artery resulted in a significant reduction of cerebral blood flow and caused severe histopathological damage in the CA1 region of the hippocampus and related behavioural deficits [56]. Daily supplementation with Tualang honey supplied efficiently improved the pathological alterations of hippocampal cells at the CA1 region and minimised neuronal loss, implicating its therapeutic neuroprotective role [57]. On another note, Tualang honey with similar supplementation also provided neuroprotection to the midbrain of rats exposed to paraquat [58]. Paraquat is a dopaminergic neurotoxin that may induce Parkinson-like symptoms in both humans and experimental animals via oxidative stress-mediated cellular injuries [59]. Tualang honey supplementation at 1 g/kg/day significantly increased tyrosine-hydroxylase immunopositive neurons in the midbrain of paraquat-exposed rats, indicating its protective effect on the midbrain dopaminergic neurons [58].

Additionally, Tualang honey has also been shown to produce neuroprotective effect in a rat model of epilepsy using kainic acid. Kainic acid is a powerful neurotoxic analogue of glutamate and an agonist of kainate subtype of ionotropic glutamate receptors. Kainic acid has been extensively used to study the mechanism of excitotoxicity-induced neurodegeneration and to establish the model for epilepsy due to its ability to induce neuroinflammation and apoptosis in the brain [60]. Pretreatment with Tualang honey at 1 g/kg body weight significantly reduced kainic acid-induced neuronal degeneration in the piriform cortex of rats [61]. Although Tualang honey supplementation failed to prevent the occurrence of kainic acid-induced seizures, it did reduce the locomotor activity and hyperactivity of the rats, indicating its effectiveness in improving the status epilepticus. It was believed that the improvement of kainic acid-induced seizure in the rat was contributed by the strong antioxidant effects of Tualang honey as it attenuates lipid peroxidation and increases the total antioxidant status in the cerebral cortex [60], cerebellum, and brainstem [62]. A subsequent study discovered that Tualang honey supplementation significantly reduced neuroinflammation and apoptosis in the cerebral cortex of the kainic acid-induced status epilepticus, as indicated by reduced levels of tumour necrosis factor-alpha (TNF-α), interleukin-1β (IL-1β), glial fibrillary acidic protein (GFAP), allograft inflammatory factor 1 (AIF-1), cyclooxygenase-2 (COX-2), and caspase-3 activity [63]. Gallic acid has been demonstrated to decrease reactive oxygen species (ROS) production, lipid peroxidation, caspase-3 activity, COX-2 expression, and prostaglandin E2 (PGE2) production in kainic acid-induced PC12 cells [64]. Gallic acid also prevents Aβ25−35 from inducing the apoptotic death of cortical neurons in vitro by inhibiting glutamate release, ROS production, and calcium release [65]. Therefore, it is postulated that the neuroprotective and antiapoptotic properties of Tualang honey may be exerted by gallic acid and possibly other polyphenolic compounds, as well as the synergistic effect of bioactive compounds. The aforementioned studies suggest that Tualang honey has neuroprotective as well as neurotrophic activities. A summary of the nootropic effects of Tualang honey in animals and humans is presented in Table 2. 

**Figure 2:**
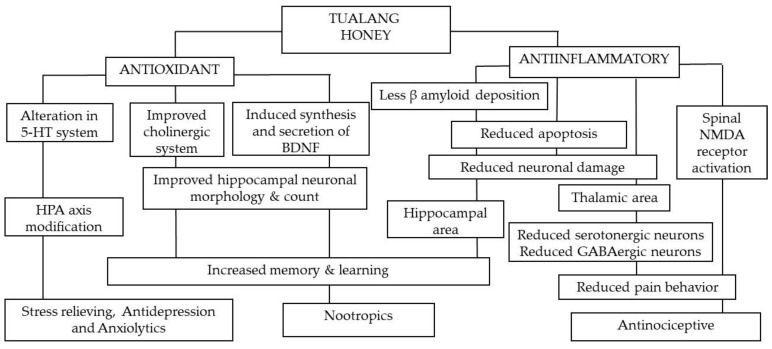
The putative neuroprotective mechanism of Tualang honey. Tualang Honey can strengthen the cellular antioxidant defence system and prevent neuroinflammation. Both antioxidant and anti-inflammatory contributed to the nootropics effects and antinociceptive effects while antioxidant is a major contributing factor to stress-relieving, antidepression and anxiolytics effect.

The mechanisms underlying the nootropic effects of Tualang honey in the above studies are illustrated in Figure 2. Tualang honey improves the antioxidant system, thus enhancing the morphology of the brain, reducing neurodegeneration, and thereby improving cognition.

### 2.2. Antinociceptive Effects of Tualang Honey

In a clinical study, it was demonstrated that patients who underwent tonsillectomy and received topical applications (2–3 mL on tonsillar fossa intraoperatively), as well as oral consumption of Tualang honey combined with sultamicillin antibiotic (4 mL honey three times daily for seven days with sultamicillin intravenously for 1–2 days and 3–7 days orally), experienced rapid pain relief during the early postoperative period and a week after tonsillectomy [66]. These patients showed minimal pain scores through the lower frequency of nighttime awakening and had reduced use of pain killers compared to patients who received sultamicillin alone. The authors postulated that Tualang honey improved post-tonsillectomy pain in patients treated with antibiotics through its soothing effects on the mucosa. A similar soothing effect of Tualang honey was also reported by Imran et al. (2011) [67] on patients with postoperative healing from split-skin graft donor sites. The application of Tualang honey hydra-gel dressing successfully aided in the healing process with minimal pain, discomfort and pruritus experienced by the patients. Moreover, the soothing effects of Tualang honey also protected the wound from evaporative water loss and dehydration, making the changing of dressing in patients easier since the dressing did not adhere to the wound. In another clinical study, newborns receiving 2 mL of Tualang honey supplementation showed similar effectiveness compared to 24% sucrose, the common solution used during neonatal venipuncture in clinical settings as revealed by the similar duration of crying time post-venipuncture (5.5 s for honey and 4 s for 24% sucrose) and premature infant pain profile score (6.5 for honey and score 7 for 24% sucrose) [68]. This study suggested that Tualang honey could be recommended as an alternative sweet substance for analgesia to be given to the newborn during venipuncture.

Studies in animals provide the means to explore the underlying mechanism for the analgesic and antinociceptive effects of Tualang honey. In a phasic pain model using the tail-flick reflex in rats, preemptive administration of Tualang honey at moderate (1.2 g/kg) and higher dose (2.4 g/kg) increased the latency of the tail-flick reflex and its antinociceptive effect was comparable to rats treated with an anti-inflammatory drug, prednisolone (10 mg/kg) [69]. When given with a tonic induction of pain using formalin injection, Tualang honey showed potent antinociception at an optimal oral dose of 1.2 g/kg body weight [70,71]. These results suggest that Tualang honey could significantly implicate pain mechanisms not only at the peripheral level but also at the central level, i.e., in the spinal cord and brain. Moreover, its antinociceptive effect was found to be more effective than vitamin C (20 mg/kg) as revealed by the lower pain behaviour responses in another study that also used formalin-induced inflammatory rat model [72]. Taken together, these investigators propose that the analgesic and antinociceptive effects of Tualang honey are attributed to its action on opioid receptors in the spinal cord and its interaction at the level of glutamate receptors in the central nervous system [73]. 

The antinociceptive effect of Tualang honey could be derived from its constituents such as gallic acid, as gallic acid attenuates nitric oxide synthase, COX-2, histamine, and pro-inflammatory cytokines released by macrophage [74]. Moreover, the anti-inflammatory properties of Tualang honey should also facilitate its antinociceptive effects as it promotes the extravasation of neutrophils at the injured site and reduces pro-inflammatory cytokines (e.g., interleukin 6) in blood serum during the course of inflammatory pain [73]. Compared to vitamin C supplementation, the effectiveness of Tualang honey in minimizing pain responses is possibly contributed by its antioxidant effects as it increases the activity of serum catalase (116.11 ng/mL in honey-supplemented rat compared to 82.05 ng/mL in control rat) and superoxide dismutase (SOD) (94.91 pg/mL in honey-treated rat compared to 46.76 pg/mL in control rat) [73]. The antioxidant properties contribute to the overall antinociceptive and antidepressant effects of Tualang honey (Figure 2). 

In another pain model combined with exposure to chronic stress, Abd Aziz and colleagues revealed that male offspring of prenatally stressed dams supplemented with Tualang honey at 1.2 g/kg for 10 days displayed increased latency of the tail-flick reflex and reduced formalin-induced pain behaviour, suggesting modulation at both peripheral and central levels [73,75]. In comparison, dams with exposure to chronic stress produced offspring with increased nociceptive behaviour [75]. This study demonstrated that supplementation with Tualang honey during pregnancy has increased the offspring’s pain threshold to phasic as well as tonic pain. Prenatal stress has been shown to result in hypercortisolemia, leading to alterations in the brain including changes in endocannabinoid levels in the hippocampus and production of oxidative stress [76]. These changes contribute to neuronal loss in the brain of offspring during fetal development [77]. Tualang honey has been found to reverse these deleterious effects on the brain as demonstrated by improvement in neuronal morphology and oxidative stress parameters such as malondialdehyde (MDA) (5.184 ng/mL in honey-treated stress group; 5.466 ng/mL in stress group), glutathione (GSH) (5.705 µg/mL in honey-treated stress group; 4.753 µg/mL in stress group) and catalase activities (10.3 ng/mL in honey-treated stress group; 8.55 ng/mL in stress group) of the prenatally stressed offspring [73]. The increased pain responses in the adult offspring of prenatally stressed mothers could result from reduced descending serotonergic neurons and local spinal cord ɣ-aminobutyric acidergic (GABAergic) inhibitory neurons, as well as the elevated activity of N-methyl-D-aspartate (NMDA) receptor [77,78]. The flavonoid in Tualang honey possibly halts the increased nociception by modulating the signalling cascades and gene expressions that participate in the transduction of pain. Subsequently, these effects avert the serotonergic and GABAergic sensory neuronal damage and downregulate the NMDA receptor activity in the CNS of the rat offspring [73]. These findings suggest that Tualang honey implicates the fetal neural development involving the modulation of nociceptive responses in the offspring in later life. Findings from studies of Tualang honey in both human and animal models present promising effects as an antinociceptive agent, as listed in Table 3. 

### 2.3. Stress-Relieving Effects of Tualang Honey

Asari et al., (2019) [79] showed that male rats induced with chronic stress had reduced corticosterone levels (24.5 ng/mL) following oral administration of Tualang honey. Acute and chronic stress may abnormally modify the cytokines level such as increased tumour necrosis factor-α (TNF-α) and IL-6, as well as decreased interferon-γ (IFN-γ) at peripheral and central levels [79]. Consequently, these alterations may increase the risk of getting cardiovascular, neurodegenerative, and autoimmune diseases. Tualang honey successfully lowered these proinflammatory cytokines in the rat brain exposed to chronic stress [79]. In another study, Haron et al. (2014) [80] demonstrated that supplementation of Tualang honey similarly reduced serum corticosterone levels in pregnant rats exposed to chronic stress (3.0 ng/mL from 4.5 ng/mL in stress group). Tualang honey might have partly minimized the adverse effects of stress on the thickness of zona fasciculata of the adrenal glands by reducing the occurrence of lipid peroxidation in the adrenal glands of the prenatal stress rat. Additionally, adverse pregnancy outcomes of the prenatally stressed rat, including a lower gestational period, were reduced after administration of Tualang honey, possibly due to its phytoestrogen effects [75].

### 2.4. Antidepressive Effects of Tualang Honey

Ovariectomised rats with depressive-like states demonstrated reduced depressive-like behaviour following the oral administration of Tualang honey, as manifested by reduced mean immobility time and increased mean swimming time [81]. After menopause, prolonged ovarian hormone deprivation may augment the effects of chronic unpredictable stress on depressive-like behaviour [82]. Besides, the ovariectomy causes a lower level of BDNF mRNA in the hippocampus of the rat [81,83]. Following supplementation with Tualang honey, the ovariectomised rat exposed to stress showed an increment in the brain BDNF concentration level (1.2 pg/mL) [81]. The antidepressant effect of Tualang honey is believed to be associated with its phytoestrogen properties, attributed to a high flavonoid content that possibly restores the HPA axis and improves mood in this rat model, hence preventing the depressive symptoms in the postmenopausal period.

Likewise, supplementation of Tualang honey for 35 days at 0.2 g/kg dose prominently counteracted the depressive-like behaviour induced by noise stress in male rats as revealed in the assessment of forced swim test [84]. Following sufficient exposure to noise stress, the HPA axis may be physically affected and accordingly contribute to acute or chronic homeostasis imbalance. The altered level of corticosterone and vasopressin may modulate behavioural despair [85]. Apart from that, the lowered activities of antioxidant enzymes followed by elevated oxidative indices in the brain indicate that oxidative stress could be the underlying factor in the pathomechanism of depression in this rat model. Supplementation with Tualang honey seems to mediate the antidepressant-like effects as it improves depressive-like behaviour, such as increased climbing and swimming times with reduced immobility times [41]. Azman and colleagues (2019) [84] agreed that Tualang honey possibly restores the HPA axis by increasing antioxidant activities including SOD, glutathione peroxidase (GPx), glutathione reductase (GR) and total antioxidant status (TAS), as well as ameliorating oxidative stress markers through reduction of protein carbonyl and MDA activities in the rat’s brain. Moreover, Tualang honey counteracts the increased level of corticosterone and ACTH to normal levels following the termination of stress. Since Tualang honey possesses several antioxidant contents, such as flavonoids as well as enzymatic and non-enzymatic substances, these constituents possibly contribute to its antidepressant effects in the depressive rat model.

### 2.5. Anxiolytic Effects of Tualang Honey

The first study investigating the anxiolytic effect of Tualang honey was on ovariectomised rats exposed to stress. This study revealed that Tualang honey increased the number of rearing events and locomotive activity, reduced mean freezing and grooming time, and decreased the autonomic nervous system response, indicating an improvement of anxiety-like behaviour [86]. The anxiolytic effect of Tualang honey could be attributed to a decrease in the brain oxidative stress that consequently modulates the brain 5-hydroxytryptamine system (Figure 2). As various phenolic acids/flavonoids were discovered in Tualang honey, such as gallic acid, kaempferol, naringenin, luteolin, syringic acid, p-coumaric acid, hyacinthim, trans-cinnamic acid, and caffeic acid, the cumulative antioxidant properties help in modifying and reducing anxiety-like behaviour [81]. As similarly reported by Azman et al. (2019) [84], Tualang honey supplementation elevated the mean activity or level of GR, glutathione S-transferases (GST), and total antioxidant capacity (TAC), and decreased the mean activity or level of oxidative stress markers, such as protein carbonyl and MDA, in the brain of the ovariectomised rat. Since postmenopausal women are prone to anxiety due to the deprived source of oestrogen, Tualang honey has the potential to act as an alternative anxiolytic agent, as its effects are comparable to those of oestrogen as demonstrated in an animal study [25,34].

## 3. Materials and Methods

For this systematic search, we developed a search strategy to identify relevant works of literature. This search strategy was tailored to four databases: Scopus, PubMed, Springer link, and Google Scholar, restricted to English articles and the following search terms were used:

(“Tualang honey” AND (memory OR pain OR stress OR antinociceptive OR antidepressant OR anxiety-like OR stress OR cerebellum OR midbrain OR cognitive OR neurodegeneration)).

**Figure 3:**
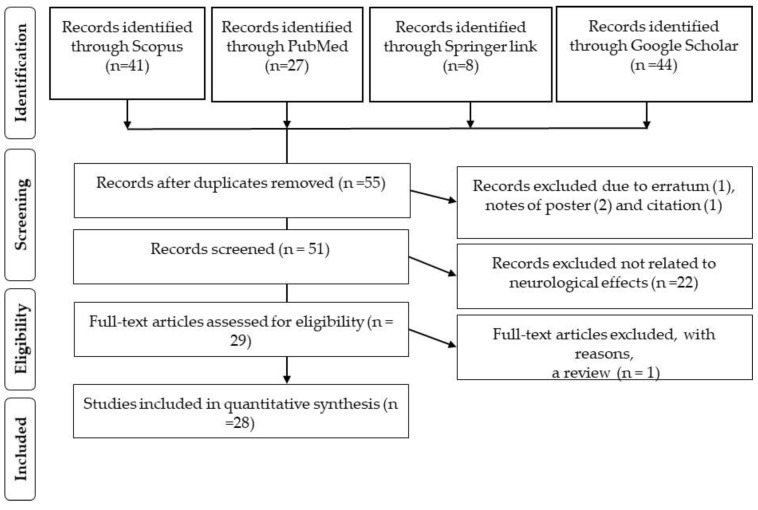
Literature inclusion and exclusion criteria at every stage (PRISMA statement).

The first study on Tualang honey in the human neurological system was conducted in 2011 [21]. Thus, the search span was from the year 2011 until 2020. A total of 41, 27, 8 and 44 records were extracted from Scopus, PubMed, Springer link database, and Google Scholar, respectively and 65 documents were excluded at this stage due to duplication. Only original articles published in journals were included, and documents from erratum (1 document), review (1 document), and note (3 documents), as well as those without neurological reports (22 documents) were excluded. We finally selected 28 articles after assessing each article using the inclusion and exclusion criteria shown in Figure 3 (PRISMA statement).

## 4. Conclusions

Our review reports the possible neurological mechanisms of Tualang honey pertaining to its antioxidant and anti-inflammatory properties. These findings could aid in the development of new therapeutic roles for Tualang honey, such as in multiple sclerosis, amylotropic lateral sclerosis, and Parkinson’s disease, as well as in determining how to get the most out of this brain supplement. In order to develop this new prospective quality standard, more research is needed to describe Tualang honey’s bioactive chemicals, molecular mechanisms, and critical components that affect nootropic action. Furthermore, proper apicultural techniques should be promoted, particularly in regions rich in tropical rain forests.

## Figures and Tables

**Table 1:** Summary of the physiochemical characteristics of Tualang honey versus Manuka honey [12].

Physiochemical Properties	Tualang Honey	Manuka Honey
Appearance	Dark brown	Light to dark brown
Specific gravity	1.34	1.39
pH	3.6–4.0	3.2–4.2
Moisture content	23.30%	18.70%
Total reducing sugars	67.50%	76.00%
Fructose	29.60%	40.00%
Glucose	30.00%	36.20%
Sucrose	0.60%	2.80%
Maltose	7.90%	1.20%
Potassium	0.51%	1.00%
Calcium	0.18%	1.00%
Magnesium	0.11%	1.00%
Sodium	0.26%	0.0008%
Carbon	41.58%	-
Oxygen	57.67%	-

**Table 2:** Summary of nootropic effects of Tualang honey on humans and animal models.

Study Model	Subject	Dose, Method of Administration and Duration of Tualang Honey Supplementation	Findings	Reference
Humans	Postmenopausal women (n = 102)	20 g/day, oral, 16 weeks	Improved verbal learning and immediate memory performance in honey-treated participants comparable with oestrogen and progestin therapy	[21,22,23]
	Schizophrenia patients (n = 80)	20 g/day, oral, 8 weeks	Improvement in total learning score across domains in immediate memory using MVAVLT in honey-treated schizophrenic patients	[24]
Animal models	Ovariectomised Sprague Dawley Rats (n = 10 per group)	200 mg/kg/bwt, oral, 18 days	Improved short term and long-term memory in Tualang honey-treated comparable to oestrogen-treated in ovariectomised rats exposed to social instability stress	[34]
	Young and aged male Sprague Dawley Rats (n = 12 per group)	200 mg/kg/bwt, oral, 28–35 days	Improved short- and long-term memory function in aged rats exposed to loud noise stress treated with Tualang honey compared to untreated rats	[39]
	Young and adult male Sprague Dawley rats (n = 12 per group)	70% honey concentration, forced feeding, 12 weeks	Improved spatial memory performance in honey-treated rats compared to untreated rats	[44]
	Adult male Sprague Dawley Rats (n = 12 per group)	200 mg/kg/bwt, oral, 14 days	Tualang honey pre-treatment showed protective effects against hypoxia-induced memory deficits compared to untreated rats	[47]
	Adult male Sprague Dawley Rats (n = 18 per group)	200 mg/kg/bwt, (methanolic fraction MTH 150mg/kg), IP, 14 days	Tualang honey and MTH improved spatial and recognition memory in LPS-induced memory deficits comparable to memantine	[51]
	Chronic cerebral hypoperfusion male Sprague Dawley Rats (n = 10 per group)	1.2 g/kg, oral, 10 weeks	Improved spatial memory performance in honey-treated cerebral hypoperfusion rats compared to untreated rats	[57]
	Adult male Sprague Dawley Rats (n = 18 per group)	TH pre-treatment (1.0 g/kg bwt) five times every 12 h	Improvement in locomotor activity in kainic acid-induced rats pre-treated with TH compared to without TH	[61]

Notes: bwt: body weight; MVAVLT: Malay Version of Auditory Verbal Learning Test; LPS: lipopolysaccharide; MTH: methanolic fraction of Tualang honey; KA: kainic acid.

**Table 3:** Antinociceptive effects of Tualang honey supplementation on human and animal models.

Study Models	Subject	Dose, Method of Administration and Duration of Tualang Honey Supplementation	Findings	Reference
Humans	Patients (3–18 y/o) underwent tonsillectomy (n = 38 each group)	Topical 2–3 mL Tualang Honey (applied on both tonsillar bed by a 3 mL syringe) + 4 mL Tualang honey three times daily for 7 days	Early postoperative pain was relieved slightly faster in Tualang honey and antibiotic group compared to the antibiotic only group	[66]
	Patients (13–65 y/o) underwent skin grafting (n = 35)	Honey hydrogel (Tualang honey was added to a mixture of 15% polyvinyl pyrrolidone (Kollidon 90), 1% protein-free agar (Oxoid) solution and 1% polyethylene glycol)	Tualang honey hydrogel may be effective in the treatment of split-skin graft donor sites with minimal pain, discomfort and pruritus.	[67]
	Neonates more than 37 weeks gestation, birth weight more than 2.5 kg, (n = 78)	2 mL of Tualang honey, oral, blinded sampling, pre-packed in 3 mL syringe, administered directly onto dorsum of infants tongue over 30 secs duration of procedure (during venepuncture)	Tualang honey was effective in relieving venepuncture pain compared to 24% sucrose	[68]
Animal models	Adult male Sprague Dawley rats (n = 24)	0.2, 1.2 or 2.4 g/kg, oral, 5 and 10 days	Preemptive administration of Tualang honey 1.2 g/kg for 5 days and 1.2, as well as 2.4 g/kg for 10 days, had a reduction in the pain behaviour score comparable to prednisolone in formalin-induced rats	[69,70,71,72]
	Male rat offsprings (n = 24)	1.2 g/kg, oral, 3 weeks	Tualang honey treated group had a significant reduction in the formalin test score in phase 1 and phase 2 compared to the stressed only group.	[73,75]

## Data Availability

Not applicable.
